# Advances in 3D and 4D Printing of Gel-Based Foods: Mechanisms, Applications, and Future Directions

**DOI:** 10.3390/gels11020094

**Published:** 2025-01-27

**Authors:** Zhou Qin, Zhihua Li, Xiaowei Huang, Liuzi Du, Wenlong Li, Peipei Gao, Zhiyang Chen, Junjun Zhang, Ziang Guo, Zexiang Li, Baoze Liu, Tingting Shen

**Affiliations:** 1 Agricultural Product Processing and Storage Lab, School of Food and Biological Engineering, Jiangsu University, Zhenjiang 212013, China; 19599960979@163.com (Z.Q.); huangxiaowei@ujs.edu.cn (X.H.); duliuzi@163.com (L.D.); 18896656873@163.com (W.L.); 2112418178@stmail.ujs.edu.cn (P.G.); junjun_5457@ujs.edu.cn (J.Z.); 18021687261@163.com (Z.L.); liu_baoze080@163.com (B.L.); shentingtingstt@ujs.edu.cn (T.S.); 2 International Joint Research Laboratory of Intelligent Agriculture and Agro-Products Processing, Jiangsu Education Department, Zhenjiang 212013, China; 15370988685@163.com; 3 College of Food Science and Engineering, Shandong Agricultural University, Tai’an 271018, China; 2112018015@stmail.ujs.edu.cn

**Keywords:** gels, customized, nutritional, food 3D printing, 4D printing

## Abstract

This review examines recent advancements in gel-based 3D and 4D food-printing technologies, with a focus on their applications in personalized nutrition and functional foods. It emphasizes the critical role of tunable rheological and mechanical properties in gels such as starch, protein, and Pickering emulsions, which are essential for successful printing. The review further explores 4D food printing, highlighting stimuli-responsive mechanisms, including color changes and deformation induced by external factors like temperature and pH. These innovations enhance both the sensory and functional properties of printed foods, advancing opportunities for personalization. Key findings from recent studies are presented, demonstrating the potential of various gels to address dietary challenges, such as dysphagia, and to enable precise nutritional customization. The review integrates cutting-edge research, identifies emerging trends and challenges, and underscores the pivotal role of gel-based materials in producing high-quality 3D-printed foods. Additionally, it highlights the potential of Pickering emulsions and lipid gels for expanding functionality and structural diversity. Overall, this work provides a comprehensive foundation for advancing future research and practical applications in gel-based 3D and 4D food printing.

## 1. Introduction

Food 3D printing is an innovative manufacturing method [1] that integrates computer software modeling and material processing technology. It has diverse applications in personalized nutrition [2], artistic food design, and customized dietary products [3]. Unlike 3D printing, 4D printing introduces the ability of printed structures [4] to adapt dynamically to external stimuli such as temperature, humidity, or pH. This adaptability enhances the sensory and functional properties of printed food products [5], catering to consumer preferences and environmental conditions. Gel materials are among the most often used raw materials in food 3D printing [6], with their properties significantly affecting the quality of the final products.

**Figure 1:**
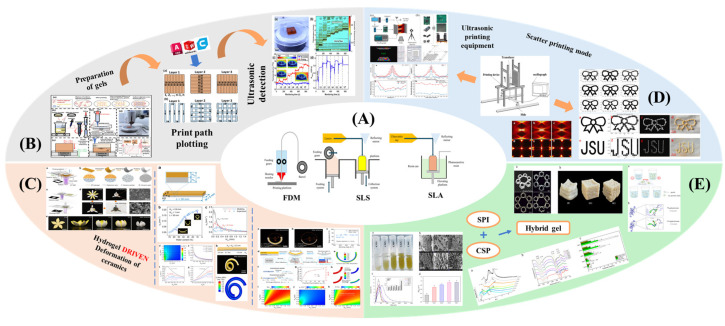
Common ways to 3D print gel food and related latest research results. (**A**) Schematic of common 3D printing process types (FDM, SLS, and SLA). (**B**) Real-time in situ ultrasound monitoring with hydrogel 3D printing. (**C**) Direct 4D printing of ceramics driven by hydrogel dehydration. (**D**) Contactless printing of food micro-particles controlled by ultrasound. (**E**) Development of soy protein isolate–chelator soluble pectin composite gels as extrusion-based 3D food printing inks: effects of mingling strategy.

Recent advances in gel-based 3D printing have led to the development of several innovative applications (Figure 1). These include real-time in situ ultrasound monitoring during hydrogel 3D printing (Figure 1B) [6], direct 4D printing of ceramics via hydrogel dehydration (Figure 1C) [7], ultrasound-contact printing of food particles (Figure 1D) [8], and chemical synthesis of soybean isolates (Figure 1E) [9]. Starch-based gels, known for their viscoelastic and structural properties [10], are widely used. The selection and preparation of gel materials must be rigorous to ensure optimal printability, structural integrity, and post-printing stability. Response surface optimization (RSO) is a frequently employed technique for optimizing gel materials [11]. Qin et al. used RSO to identify the ideal starch gel formulation by adjusting parameters such as water content, amylopectin-to-amylose ratio, and printing speed, achieving high precision and structural quality [12].

Research on 4D printing of food products primarily focuses on two areas: color change and deformation [13], both of which enhance visual appeal and product value [14]. Color change can be induced through water electrolysis, creating pH gradients at the electrodes that activate pH-sensitive pigments in the printing ink. The process is controlled by modulating voltage and current waveform. Deformation studies often involve high-temperature baking, where dehydration causes structural changes in printed foods. These dynamic transformations demonstrate the potential of 4D printing to create interactive food designs, opening avenues for innovation and consumer engagement [15].

**Figure 2:**
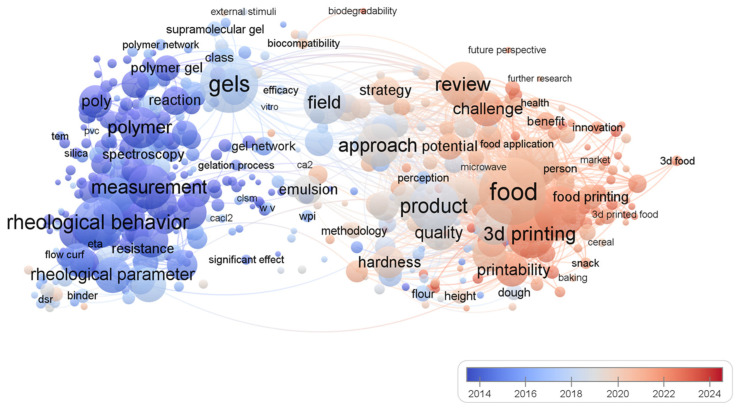
VOS viewer-based network map of hotspots and trends co-occurring in food 3D printing and gelation research.

Figure 2 illustrates the research hotspots and trends in “food printing” and “gels”. Early studies (blue area, ~2014) focused on the rheological and material properties of gels, such as “shear rate”, “yield stress”, and “polymer”, highlighting the foundational role of rheology and material science in the field. Recent studies (red area, ~2024) have emphasized the application and advancement of food 3D printing, with keywords like “food printing”, “3D printing”, “technology”, and “nutrition”, indicating a shift toward technical implementation, product development, and nutritional considerations. The network structure reveals a close relationship between material properties and printability, underscoring the critical role of rheological properties in 3D printing quality. Additionally, keywords such as “sustainability” and “technology” suggest future directions for optimizing food printing techniques and promoting sustainable applications. This field is evolving from foundational studies in rheology and materials toward technological innovation and interdisciplinary applications in food printing.

This review highlights the core aspects of gel-based food 3D printing technology and examines the role of functional gel-based 3D-printed foods enriched with nutritional components. It discusses commonly used gels such as starch, surimi, and protein gels, as well as specialized gels like Pickering emulsions, lipid gels, and polysaccharide gels, which require unique processing methods. The mechanisms through which functional substances within these gels exert their effects are also explored. Additionally, this paper addresses how the distinctive rheological and textural properties of gel-based 3D-printed foods can cater to the specific needs of individuals with dysphagia and support the growing demand for personalized and customized nutritional foods. It provides an overview of advancements in 4D-printed functional foods and proposes potential directions for future developments in gel-based 4D food printing. The objective of this review is to inspire enthusiasm and foster interest in the development and application of gel-based 3D and 4D food-printing technologies within the broader context of health-conscious dietary trends, encouraging innovations that promote individual well-being and nutrition.

## 2. Functional Gels in Food 3D Printing

### 2.1. Methods

The study of 3D printing with food gels involves a range of methodologies. First, suitable base materials are identified through the selection and evaluation of gel types, such as starch, proteins, or Pickering emulsion gels. Next, gel formulation optimization is conducted to ensure desirable properties, including extrusion performance, shape retention, and printing precision. To assess the physical and functional attributes of printed products, rheological tests are employed to analyze the flow behavior and viscoelasticity of the gels, while texture analysis evaluates the mechanical strength and structural integrity of the final products. Scanning electron microscopy (SEM) is used to examine the microstructure of the samples, providing insights into the effects of the printing process on internal organization. For 4D printing, specialized treatments (e.g., color change or deformation) are applied to evaluate the materials’ responsiveness to external stimuli such as temperature, humidity, or pH changes. These combined methods offer a comprehensive framework for advancing the development and application of 3D and 4D printing technologies in food gels.

### 2.2. Starch Gel

Starch, as the main source of carbohydrates, is predominant in grains such as wheat, corn and potatoes and is an integral part of the daily diet [16]. The composition of grain starch also includes moisture, proteins, fats, and sugars [17,18], which directly affect its physicochemical properties, rheological characteristics, and pasting and aging processes [19]. Starch gels are based on semi-crystalline starch granules that change from an ordered to a disordered structure when heated in water, a process known as pasting [20]. The pasted starch shows great potential for application in 3D printing, especially in the ability to be effectively extruded and layered under high shear printing conditions [21]. Starch pasting is accompanied by the phenomena of solubility changes, viscosity increases, and aging, in which aging is the inverse process of water absorption and reorganization of dissolved starch molecules to form a natural starch-like structure [22]. The formation process of starch gel includes the release of amylose when starch granules absorb water and heat, as well as the entanglement of molecules to form a three-dimensional network structure. Further heating and stirring cause the remaining granules to break and enter the water phase. After cooling, amylose and amylopectin combine through hydrogen bonds, eventually forming a stable gel [23]. Some applications of starch gels in food 3D printing are described below, and their printing results are shown in Table 1, the main sources of starch are wheat, cassava, potatoes, rice and corn [24,25,26,27].

Existing research primarily focuses on optimizing factors such as moisture content and the ratio of amylose to amylopectin [28] to enhance printing performance. While extrusion printing technology has become mainstream, issues related to printing accuracy and stability remain unresolved. Moreover, the development and scale-up of functional food applications using starch gels present significant challenges [29]. Challenges in utilizing starch gels for functional food development include difficulties in optimizing rheological properties, instability in multi-component formulations, and inadequate stability and bioavailability of functional ingredients. Additionally, thermal sensitivity and moisture migration during processing often lead to structural instability. Furthermore, storage issues, such as hygroscopicity, hardening, and retrogradation, can significantly impact product quality. Addressing these challenges requires a multidisciplinary approach to enable the broader application of starch gels in functional foods. To address these issues, there is an urgent need to improve the rheological properties and gel stability of starch materials, enhance the precision and intelligence of the printing process, and develop more personalized and functional foods [30]. Future research should focus on optimizing starch material properties through multi-component synergistic enhancement and molecular mechanism studies [31], integrating multi-material printing with real-time monitoring technologies for high-precision printing, and promoting the development of green and sustainable materials for industrial applications. These efforts will help to expand the potential of food 3D printing.

### 2.3. Surimi Gel

Surimi is a low-fat food rich in high-quality protein, polyunsaturated fatty acids, minerals, and vitamin B complexes [32], which has high nutritional value and is easy to digest for the elderly, children, and other special populations [33,34]. Its applications in food 3D printing are of great value. Myofibrillar proteins in surimi help to form thermoelastic gels [35], which provide good rheological properties and self-supporting ability to adapt to the 3D printing process. Compared with traditional methods, 3D-printed surimi products are more flexible and malleable, and can precisely control the morphology and texture of food products, meet the taste needs of special groups, and open a new direction for the development of personalized and healthy food products. Table 2 shows some studies on surimi gels in food 3D printing [36,37,38,39].

### 2.4. Protein Gel

Protein gel 3D printing technology has gained significant attention in the field of food engineering in recent years [40]. Traditional food processing methods typically rely on mass production and standardized processes, which struggle to meet the growing demand for personalized and customized products [41]. As consumer expectations for functionality, nutritional value, and sensory experience increase, 3D printing has emerged as an innovative food manufacturing approach capable of addressing these needs. Protein, a key nutritional component in food, possesses excellent gelation properties, making it suitable for forming complex structures during 3D printing [42]. The primary advantage of protein gel 3D printing lies in its ability to precisely control the gelation and structuring of proteins during the printing process, enabling customized product design and offering greater creative possibilities in the food industry.

In recent years, researchers have investigated the gelation properties of various protein sources, including plant, whey, soy, and pea proteins, to assess their suitability for 3D printing [43]. They have optimized the effects of WPI, SA, and water bath heating time on 3D printed food gels to improve the gel printing performance [44]. Li et al. investigated the effects of different polysaccharides on the rheological properties, stability, and 3D printing performance of whey protein isolate emulsions [45]. After appropriate treatment, these proteins can form stable gel networks with favorable rheological properties [46], enabling stable printing in 3D printers. Research on plant proteins and soy proteins has demonstrated significant progress [47], with scientists improving gel printing performance by adjusting factors such as protein concentration, pH, salt concentration, and temperature. These optimizations have not only enhanced the appearance and texture of 3D printed foods, but also improved their nutritional value and functionality.

Despite significant progress in protein gel 3D printing technology, several technical and practical challenges remain. First, the printing stability and structural accuracy of protein gels require further optimization. In particular, the printing of high-concentration, high-viscosity protein solutions is complicated by the complex rheological properties of the gel, which can result in variations in printing precision and stability due to external factors such as temperature and humidity. Additionally, the retention of bioactivity and nutritional content during the printing process, as well as the interactions between protein gels and other food components, continue to be key areas of research. Future studies should focus on addressing these issues by improving printing processes, enhancing print quality, and ensuring that the nutritional and functional properties of printed foods are maintained.

### 2.5. Pickering Emulsion

Pickering emulsions are stabilized by solid particles adsorbed at the oil–water interface [48], offering advantages such as high safety, low cost, excellent stability, and favorable rheological properties [49,50]. These characteristics make them valuable in food, biomedicine, and cosmetics [51]. Understanding the factors influencing their stability is essential for expanding their applications. Surface-active hydrocolloids can adsorb at the oil–water interface to reduce interfacial tension, forming a protective layer that prevents droplet aggregation and improves stability [52]. This function underpins their use in food emulsions to encapsulate lipophilic compounds, delay lipid digestion, and enable targeted delivery [53]. However, the weak interfacial adsorption of some polysaccharides due to their hydrophilic nature requires modification, such as physical or chemical treatments or forming complexes (e.g., polysaccharide–protein), to enhance their emulsifying ability [54].

The stability of Pickering emulsions also relies on the dense interfacial layer formed by stabilizing particles and their interactions, such as three-dimensional networks or steric hindrance, which prevent droplet aggregation [55]. Additionally, external factors like pH, ionic strength, and oil–water ratio significantly affect stability, highlighting the need for careful formulation to optimize applications [55,56]. In recent studies, efforts have been made to further enhance the stability and performance of Pickering emulsions for specific applications. For example, Guo et al. developed Pickering emulsions to meet the requirements of food 3D printing by modulating β-CD emulsification properties via CMC, optimizing the biphasic wettability and rheological properties of interfacial films for stable printing performance [57]. Similarly, Cen et al. innovatively used β-cyclodextrin-modified citrus pectin to stabilize Pickering emulsions and optimize their dual wettability and rheological properties to meet food 3D printing requirements [58]. Looking ahead, building on these advancements, future research is increasingly shifting toward the integration of Pickering emulsions into 4D printing. For instance, Guo et al. explored temperature-driven 4D printing of Pickering emulsions, demonstrating dynamic color changes. Their work provides innovative methods and theoretical foundations for the development of colorful and personalized food applications [59].

### 2.6. Lipid Gel

Food lipid gel 3D printing has emerged as an innovative research direction in food engineering [60], aiming to create complex, customized lipid-based food structures through 3D printing [61]. Due to its excellent plasticity, thermal stability, and sensory appeal, lipid gel has become an ideal material for producing high-quality foods [62]. Studies have shown that lipid gels can be manipulated in 3D printing by adjusting their composition, structure, and processing conditions [63], allowing for precise control over the printed product’s form and functional properties, such as texture, structural strength, and nutritional content [64]. Furthermore, the application of lipid gels has expanded from traditional high-fat foods like chocolate and cream to the development of low-fat and functional foods. Table 3 lists some studies on lipid gels, mainly focusing on chocolate [65,66,67] and cheese [68,69].

However, several limitations remain in the current research on food lipid gel 3D printing. First, the rheological properties and printing stability of lipid gels are key factors influencing print quality [60], especially since they are highly sensitive to external environmental factors such as temperature and humidity. Additionally, the long-term stability of lipid gels and their compatibility with other food ingredients require further investigation to ensure quality during storage and processing [63,70]. Furthermore, the development of accurate modeling and simulation techniques to optimize lipid gel printing performance, particularly in large-scale production, remains a significant challenge.

Future research should focus on optimizing lipid gel formulations and processing techniques to improve their flowability and structural stability during 3D printing [60]. Multi-scale modeling and advanced simulation technologies can be employed to explore the deformation and stability mechanisms of lipid gels under different printing conditions. Moreover, developing specialized lipid gels—such as those containing bioactive compounds or improving sensory properties through composite materials—will likely drive the advancement of lipid gel 3D printing technology in the production of personalized and nutritionally functional foods.

### 2.7. Optimizing Gel-Based Materials for Enhanced 3D Food Printing Performance

In 3D food printing, gel-based materials play a pivotal role in optimizing texture, structure, and nutritional properties. Each type of gel brings unique functional advantages, enabling diverse applications in the food industry [71]. Starch gels are widely recognized for their excellent printability [72], forming stable gels with adjustable viscosity that support the creation of complex shapes. Their gelation process is driven by water absorption and the formation of cohesive networks [73,74], which enhance structural integrity. Surimi gels, commonly used in seafood printing, achieve a soft, chewy texture through the addition of stabilizers such as alginate or gellan. These gels rely on heat or ionic crosslinking to balance strength and elasticity, making them particularly suitable for specialized applications like foods designed for individuals with swallowing difficulties [75]. Protein-based gels, derived from sources like egg whites, whey protein, or plant-based proteins, exhibit strong gelling properties due to protein unfolding and bond formation under heat [76]. These gels not only deliver high nutritional value, but also provide functional flexibility for health-conscious consumers, including allergen-free or vegan options. Lipid gels, formed by combining oils or fats with gelling agents like monoglycerides or lecithin, contribute to a rich, smooth texture and enhance mouthfeel [77]. Their controlled cooling-induced solidification makes them ideal for encapsulating flavors or bioactive ingredients, supporting indulgent or functional food designs [78]. Finally, Pickering emulsions, stabilized by particles such as starch or proteins, create high-viscosity gels with excellent stability. These emulsions enable the printing of both rigid and flexible structures, supporting intricate textures and multi-layered designs [79].

To maximize the potential of gel-based materials in 3D food printing, targeted improvements are essential. Starch gels can benefit from modifications to enhance rheological properties and stability [80], while fish paste gels require advancements in gelling agents and crosslinking methods to improve elasticity and strength for foods targeting individuals with swallowing difficulties. For protein gels, developing plant-based options that meet allergen-free or vegan demands is critical [81]. Pickering emulsions could be further optimized with novel particles and multi-layered printing techniques for enhanced stability and functionality. For lipid gels, research should focus on incorporating functional oils to improve texture and stability. Together, these innovations will push the boundaries of 3D food printing, expanding its applications in texture customization, personalized nutrition, and functional food development, ultimately driving technological and commercial advancements in the food industry.

## 3. Market Application and Development

The application of 3D food printing technology, particularly in the context of food gels [82], offers significant benefits for individuals with swallowing difficulties. This technology enables the precise control of food texture, shape, and nutritional content, addressing the specific dietary needs of this population [83] and mitigating the health risks associated with dysphagia. Through 3D printing, food gels can be customized to achieve optimal texture and structural integrity [84], enhancing both safety and ease of consumption. Additionally, the technology allows for the precise formulation of nutrients tailored to individual health requirements, which is particularly advantageous for special populations such as children, the elderly, and individuals with medical conditions that require dietary restrictions. As such, 3D printing of food gels offers a promising approach to personalized nutrition [85,86], not only improving the safety and palatability of meals but also contributing to the overall well-being and quality of life of these target groups.

### 3.1. Suitable for People with Swallowing Difficulties

The development of 3D printing technology for gel-based foods holds significant potential for addressing the needs of individuals with swallowing difficulties [87,88]. This population requires foods with specific textures, shapes, and nutritional profiles to mitigate the health risks associated with dysphagia [89]. Traditional culinary tools are limited in their ability to create intricate shapes and designs [90], whereas 3D printing technology offers unparalleled advantages in shape customization and personalized nutritional enhancement. By optimizing printing materials and processes, it is possible to design gel-based foods with desirable textures and balanced nutritional content. Zhu et al. functionalized surimi using beet glycoside/gelatin/nano chitin complexes and investigated its application in 4D printing and dysphagia diets [91]. They also investigated the optimization of the texture, stability, and swallowing safety of 3D-printed pea isolate protein products by physical modification [92]. The development of dysphagia diets using 3D printing technology optimizes colloidal formulations and enhances antioxidant capacity and bioavailability [93]. These advancements not only meet the dietary requirements of individuals with swallowing disorders, but also enhance the visual appeal and sensory experience of the foods, providing innovative solutions for specialized dietary needs.

### 3.2. Precise Nutritional Customization

The emerging technology of 3D food printing using gels demonstrates significant potential in precision nutrition and customized dietary solutions [94]. Precision nutrition aims to provide tailored nutritional formulations based on individual physiological characteristics and health needs, and 3D printing technology offers a reliable platform to achieve this goal [40,95]. By precisely controlling the composition and structure of foods, gel-based 3D printing enables the creation of personalized foods tailored to an individual’s age, gender, weight, or specific health requirements [96], such as managing diabetes or heart disease. This capability is particularly relevant in addressing the nutritional needs of aging populations and patients with chronic illnesses [97].

Three-dimensional food printing excels by adjusting ingredient ratios, printing parameters, and gel material properties [98] to ensure the consistent distribution of nutrients within food structures [99]. Compared to traditional food processing methods, 3D printing offers greater flexibility in modifying physical and chemical properties. Zhang et al. optimized buckwheat dough formulation for enhanced 3D printability while maintaining its nutritional value [31], demonstrating the technology’s ability to align food properties with individual dietary needs. Wang et al. successfully developed 3D-printed oral drug tablets with specific sustained-release properties [100]. And research has been conducted on the potential of 3D printing (FDM and SLA) in creating personalized and flexible acne treatment devices [101]. Additionally, 3D printing can create foods with specific microstructures [102], enabling controlled nutrient release and absorption. This structural customization opens new possibilities for the development of functional foods—foods designed to provide specific health benefits [103], such as boosting immunity, improving digestion, or delivering antioxidant properties [104]. By incorporating high-fiber components, prebiotics [82], or plant extracts, 3D printing can produce personalized functional foods for different demographics [64,105], with precise control over the release and bioavailability of functional ingredients to maximize health benefits [106].

**Figure 3:**
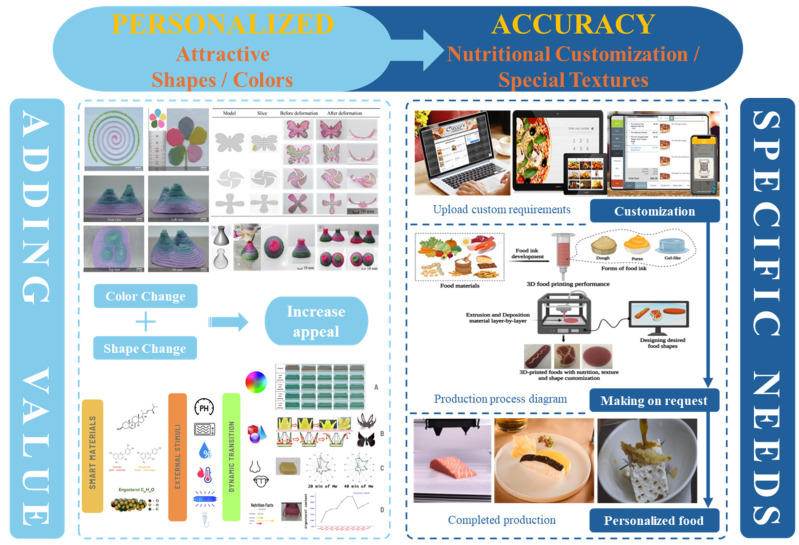
Food gel 3D printing for personalization and precision nutrition.

Gel-based 3D food printing offers transformative potential in precision nutrition, personalized dietary solutions, and functional food development. By enabling precise control over nutrient composition, food structure, and sensory attributes, the technology not only meets individual nutritional needs but also contributes to reducing food waste and advancing sustainability in the food industry (Figure 3) [20,107,108]. With continued advancements, 3D printing is poised to play a pivotal role in large-scale personalized nutrition, food industry innovation, and human health improvement. As market demand for dynamic functional foods continues to grow, 4D printing technology has emerged as a promising innovation. Unlike the static structures created through 3D printing, 4D printing incorporates the dimension of time, enabling printed foods to respond dynamically to external stimuli, such as temperature, humidity, or pH, by undergoing changes like color transformation or deformation. This technology not only expands the possibilities for food design but also offers innovative opportunities in personalized nutrition, functional foods, and interactive consumer experiences. By exploring the unique responsive mechanisms of gel-based materials in 4D printing, this approach can further advance food printing technology, transitioning it from static production to dynamic and adaptive applications.

## 4. Functional Foods with 4D Printing Technology

### 4.1. 4D Printing Concept and Status

The concept of 4D printing was first introduced by Prof. Tibbits at MIT [109]. This groundbreaking technological advancement enables 3D-printed objects to undergo programmable transformations over time, representing the fourth dimension. In food-related research, 3D food printing has garnered significant attention for its ability to produce uniquely shaped products with enhanced nutritional value and ease of digestion [13], making it a prominent research focus. A key advantage of 4D printing is its adaptability to various materials [110], enabling the customization of materials for specific applications [111]. Furthermore, the shape of food products significantly influences consumer preferences [112]; attractive and visually appealing shapes can enhance the allure of a product and increase its perceived value [113]. Four-dimensional printing is an emerging technology in the field of additive manufacturing technology that introduces the concept of changing print configurations [114], over time (including shape and color), thus extending 3D printing’s traditional capabilities [111].

Current research on 4D printing of food gels has made significant progress in exploring programmable transformations of food structures over time, enhancing their functionality and consumer appeal [115,116]. Studies have primarily focused on material formulations, including the incorporation of responsive components such as polysaccharides, proteins, and edible colorants, which enable stimuli-induced changes like shape morphing, texture variation, and color shifting [81]. Advanced techniques, such as response surface methodology and rheological characterization, have been employed to optimize printing parameters and material properties. However, several challenges remain. The limited range of food-grade responsive materials, inconsistencies in the printing and transformation processes, and the lack of standardized evaluation metrics hinder the scalability and reproducibility of these systems [13]. Additionally, ensuring the sensory and nutritional quality of printed products while maintaining cost-effectiveness poses significant obstacles to broader commercial application [117]. Further interdisciplinary research is needed to address these issues and realize the full potential of food gel 4D printing in industrial and consumer contexts [118].

### 4.2. Discoloration Printing (Electrolytic Water)

In recent years, as color saturation has increased, some commercial printers have enabled color 3D printing. Diamond Hotend produces rainbow-colored parts by mixing three types of filaments [119], and XYZprinting’s da Vinci Color uses CMKY Inkjet Cartridges for Full Color Printing [120]. Zhai et al. developed an anthocyanin-based colorimetric gas sensor with an oleogel hydrogel structure to protect anthocyanins for real-time monitoring of meat and fish freshness [121,122,123,124,125]. Anthocyanins, as natural plant pigments [126,127,128,129,130,131,132], are widely used in color-changing printing for food gels due to their sensitivity to pH variations. They can alter their color under different acidic and alkaline conditions, functioning as pH indicators to enable dynamic color changes during the printing process [133,134]. This characteristic not only enhances the visual appeal of food, but also offers new possibilities for the development of functional foods [135]. Current research primarily focuses on optimizing the stability of anthocyanins and expanding their color-changing range [136] by adjusting factors such as material composition, pH, and temperature to ensure their effective application in food printing. Furthermore, anthocyanin-based color-changing printing is also applied in areas such as smart food packaging and personalized food products. Curcumin is a flavonoid compound [137,138,139,140,141,142]. It is not only the main active ingredient of turmeric [143,144,145], but also one of its main pharmacological components [146,147,148]. It is widely used in traditional Chinese medicine and is gaining attention in modern medicine and nutrition. Curcumin and anthocyanins play similar roles in food gel 3D printing. Four-dimensional printing of citrus pectin/β-CD stabilized Pickering emulsion by addition of pH-sensitive curcumin in a study by Cen et al. [149], and a 4D printing system was stimulated by curcumin/whey isolate protein nanoparticles to enhance color change and antioxidant capacity [148]. These studies demonstrate the feasibility of applying curcumin in color-changing printing of food gels. Table 4 shows some studies on gel color 4D printing, among them are dual-nozzle food printer [114], pH-responsive color-changing composite hydrogel [150], WPI/FDRCJ/GA particle microwave-induced color change [150], infrared drying curcumin 4D printing [151], microwave-induced emulsion color change [152] and microwave-induced microcapsule 4D printing [153].

However, the long-term stability of anthocyanins and curcumin, as well as the precision of their color-changing responses in food printing, remain challenging. Future research needs to gradually address these issues.

### 4.3. Transformation Printing (Dehydration Model)

Food gel deformation in 4D printing has emerged as an innovative technique in the food manufacturing field, attracting significant attention in recent years [13]. Building on 3D printing, 4D printing adds the dimension of time, enabling printed structures to deform and adapt in response to external stimuli such as temperature, humidity, or pH changes. Food gels, known for their versatility and adjustability, are particularly suitable for 4D printing due to their favorable rheological properties and controllable gelation characteristics [154]. Research has shown that the deformation behavior of food gels, such as thermal responsiveness and shape memory [155], can be controlled by adjusting their composition, structure, and processing parameters, thereby enabling the design of personalized, custom-made food products.

However, several limitations remain in the current research on food gel deformation in 4D printing. First, the deformation performance and stability of gel materials need further optimization [156], particularly in complex and variable environmental conditions, where deformation effects and precision can be uncertain. Second, the long-term stability and safety of food gels require more thorough investigation, especially concerning food preservation, nutrient retention, and sensory characteristics. Moreover, accurately predicting the deformation behavior of food gels under external stimuli using effective modeling and simulation techniques remains a significant challenge.

Future research should focus on optimizing the formulation and processing of food gel materials to enhance deformation precision and response rates [42,157]. Multi-scale modeling and advanced simulation techniques could be used to explore the deformation mechanisms of food gels under different environmental conditions. Additionally, integrating smart sensors and control systems for precise real-time regulation will be crucial. Furthermore, developing more innovative functional food gel materials, such as composites that incorporate bioactive components or improve sensory properties, will be key to advancing 4D printing technology for food gel deformation. Table 5 shows some studies on gel deformation printing. The deformation strategies are mainly divided into thermal drive [158,159,160] and microwave induced [161,162].

## 5. Conclusions

With the rapid advancement of science and technology, the integration of 3D and 4D printing technologies into the food industry has gained momentum, gradually transforming traditional food processing methods. These innovations not only hold the potential to address food supply challenges, but also offer a means to optimize the nutritional composition of functional foods through precise formulations. From a cost-effectiveness standpoint, 3D and 4D printing can adapt the structure and composition of food to specific demands, maximizing the use of raw materials while minimizing waste. This paper explores the application of gel-based materials in 3D and 4D printing, focusing on the functional properties of starch, proteins, and Pickering emulsion gels, as well as their potential within the food sector.

From a market perspective, as consumer demand for personalized and nutritionally optimized foods continues to rise, 3D printing is poised to become an essential tool in the food industry. Compared to traditional food processing methods, 3D printing can reduce production costs and enhance production efficiency, particularly in the development of personalized nutrition and functional foods. Furthermore, 4D printing technology, by incorporating the dimension of time, has the potential to further enhance the sustainability and adaptability of food products.

Looking ahead, the integration of 3D and 4D printing technologies in the food industry is expected to deepen, particularly in areas such as improving production flexibility, reducing waste, and promoting sustainability. With evolving market demands and continuous technological advancements, food-printing technologies are poised to make a significant impact on personalized nutrition, functional food development, and the long-term sustainability of the food industry.

## Figures and Tables

**Table 1:** Application of starch gels in food 3D printing.

Starch Gel Source	Research Content	Printing Results	Microstructure
Wheat and cassava	Effects of pulsed electric field treatment on wheat and cassava starch properties and 3D printing applications were investigated	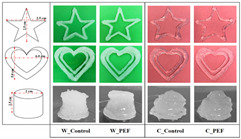	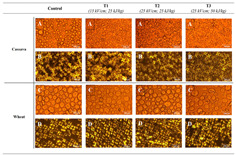
Potato, rice, and corn	The effects of the rheological properties of potato, rice, and corn starches on the feasibility of 3D printing were investigated	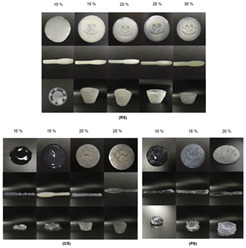	NONE
Potato	The rheological properties of potato slurry and its application in three-dimensional printing were investigated, and the correlation between formulation and printability was modeled	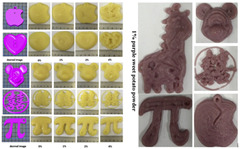	NONE
Corn	Bigels made with a high hydrogel-to-oleogel ratio have better 3D printing properties and can be successfully used as a solid fat substitute	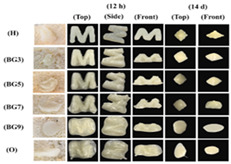	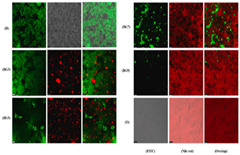

**Table 2:** Application of surimi gel in 3D printing.

Source of Surimi	Research Content	Printing Results	Microstructure
Basa fish	Exploring the use of balsa protein-stabilized Pickering emulsions in food 3D printing	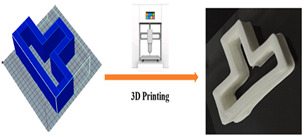	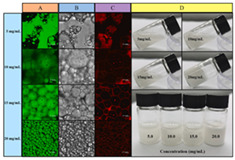
Golden thread fish	Improved molding of microwave 3D-printed surimi by using a combination of gelatin and κ-carrageenan	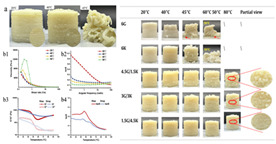	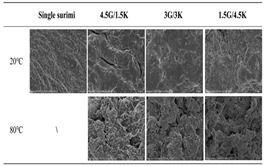
Tilapia	Discovery of Ulva polymorpha (UP) powder enhances the printability and microstructure of tilapia surimi inks	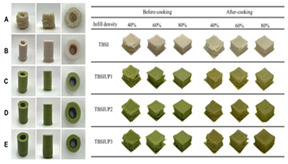	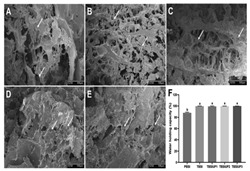
Chinese Herring	The use of vegetable oil bodies improves structural stability through emulsification, optimizes printability, and improves gel characteristics	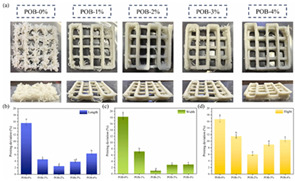	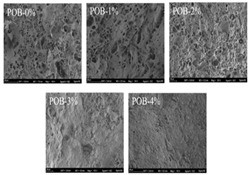

**Table 3:** State of the art in lipid gel 3D printing research.

Lipid Source	Research Content	Key Factory	Printing Results
Black chocolate	Designed a cooling system to make chocolate solidify quickly and improve printing quality	Cooling and condensation	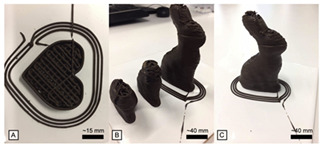
Black chocolate	Modeling optimization can effectively help with understanding the structural strength of chocolate 3D printing	Modeling assistance	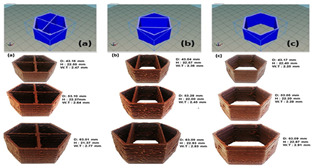
White chocolate	Comparison of 3D print processes used syringe/extruder and commercial printer, as well as analysis of force–displacement curves	Force-displacement	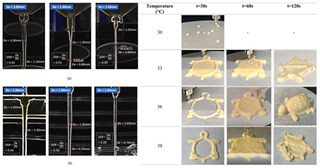
Cheese	WPNF can reduce the fat content of cheddar cheese and improve its 3D printing suitability and stability	WPNF	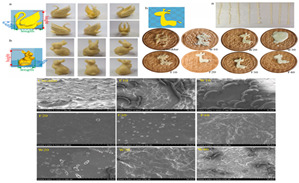
Cheese	Factors such as pH value, texture, and temperature significantly affect the 3D printing suitability of processed cheese	Effect of pH on texture	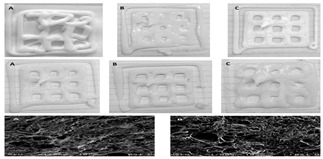

**Table 4:** Gel color change-related 4D printing research.

4D Color Change Strategy	Research Content	Printing Results	Printing Models/ Microstructure
pH response color change	Using a dual-head food printer, cone-shaped mashed potatoes with two colors per layer are printed	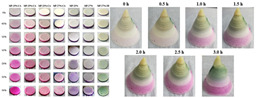	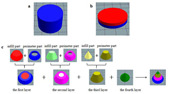
pH response color change	Development of a pH-responsive color-changing peanut protein–polysaccharide composite hydrogel	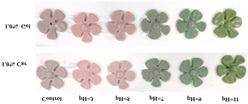	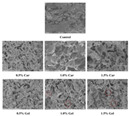
Thermal response of anthocyanins	WPI/FDRCJ/GA particles can make 3D-printed food change color quickly under microwave heating	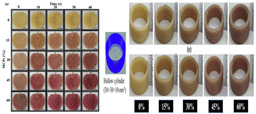	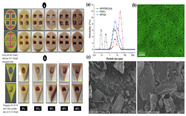
Curcumin dehydration discoloration	Improving curcumin stability and 4D printing effect through nanoembedding technology and catalytic infrared drying	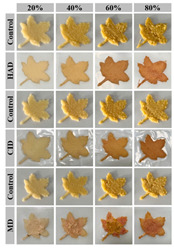	NONE
Curcumin dehydration discoloration	Microwave-induced color change of curcumin emulsion 3D-printed food	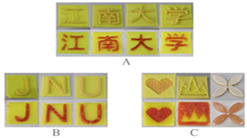	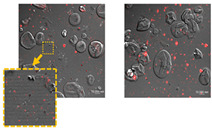
Curcumin dehydration discoloration	Microwave heating induces color and flavor changes of microcapsule 4D-printed food	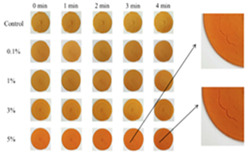	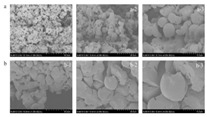

**Table 5:** Induced deformation of gels for 4D printing.

4D Deformation Strategy	Research Content	Printing Results	Microstructure/ Complex Deformation
Thermal drive	Controlled deformation of 3D printed pumpkin puree/paper bilayer structure by air-drying treatment	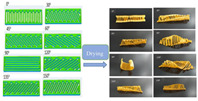	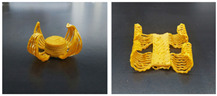
Thermal drive	Insect food is 3D-printed with edible insect ink, showing adjustable 4D deformation properties under thermal stimulation	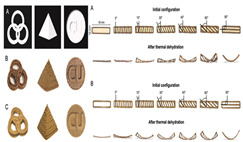	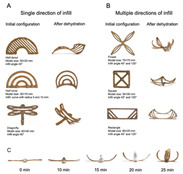
Thermal drive	Controls the continuous phase and interface, designs edible emulsion gels, and realizes 3D/4D printing and deformation	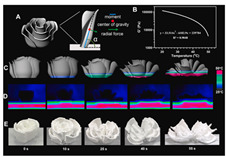	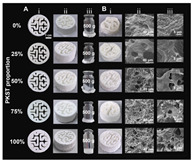
Microwave-induced	Microwave dehydration induces directional bending deformation, and the internal material affects the bending angle	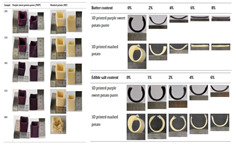	NONE
Microwave-induced	4D printing of sodium alginate and chestnut powder was achieved	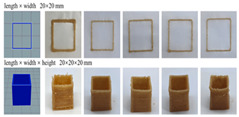	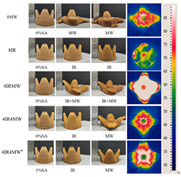

## Data Availability

No new data were created or analyzed in this study. Data sharing is not applicable to this article.

## References

[1] Dhand A.P., Davidson M.D., Burdick J.A. (2024). Lithography-Based 3D Printing of Hydrogels. Nat. Rev. Bioeng..

[2] Verma M., Hontecillas R., Tubau-Juni N., Abedi V., Bassaganya-Riera J. (2018). Challenges in Personalized Nutrition and Health. Front. Nutr..

[3] Guo Z., Arslan M., Li Z.H., Cen S.Y., Shi J.Y., Huang X.W., Xiao J.B., Zou X.B. (2022). Application of Protein in Extrusion-Based 3D Food Printing: Current Status and Prospectus. Foods.

[4] Momeni F., Liu X., Ni J. (2017). A review of 4D Printing. Mater. Des..

[5] Rastogi P., Kandasubramanian B. (2019). Breakthrough in the Printing Tactics for Stimuli-Responsive Materials: 4D Printing. Chem. Eng. J..

[6] Yang T., Jin Y., Smith L.M., Dahotre N.B., Neogi A. (2024). Real-Time In-Situ Ultrasound Monitoring of Soft Hydrogel 3D Printing with Subwavelength Resolution. Commun. Eng..

[7] Wang R., Yuan C., Cheng J., He X., Ye H., Jian B., Li H., Bai J., Ge Q. (2024). Direct 4D Printing of Ceramics Driven by Hydrogel Dehydration. Nat. Commun..

[8] Li Z., He W., Huang X., Hu X., Chen H., Zhang Y., Shi J., Zou X. (2025). Contactless Printing of Food Micro-Particles Controlled by Ultrasound. J. Food Eng..

[9] Xie J., Bi J., Liu X., Blecker C., Jacquet N., Lyu J. (2024). Development of Soy Protein Isolate-Chelator Soluble Pectin Composite Gels as Extrusion-Based 3D Food Printing Inks: Effects of Mingling Strategy. Food Hydrocoll..

[10] Shao G.-Q., Zhang H., Xu D., Wu F.-F., Jin Y.-M., Yang N., Yu K.-J., Xu X.-M. (2023). Insights into Starch-Based Gels: Selection, Fabrication, and Application. Int. J. Biol. Macromol..

[11] Bezerra M.A., Santelli R.E., Oliveira E.P., Villar L.S., Escaleira L.A. (2008). Response Surface Methodology (RSM) as a Tool for Optimization in Analytical Chemistry. Talanta.

[12] Qin Z., Li Z., Zou X., Guo Z., Wang S., Chen Z. (2024). Simulation of Starch Gel Printing and Deformation Process Using Comsol. Foods.

[13] Teng X., Zhang M., Mujumdar A.S. (2021). 4D Printing: Recent Advances and Proposals in the Food Sector. Trends Food Sci. Technol..

[14] Guo C., Zhang M., Bhandari B., Devahastin S. (2022). Investigation on Simultaneous Change of Deformation, Color and Aroma of 4D Printed Starch-Based Pastes from Fruit and Vegetable as Induced by Microwave. Food Res. Int..

[15] D’Souza C., Adkari A., Alahakoon D. (2024). Coupling AI with Empirical Research–A Case of 3D Printed Food Technology. Food Qual. Prefer..

[16] Lin D., Ma Y., Qin W., Loy D.A., Chen H., Zhang Q. (2022). The Structure, Properties and Potential Probiotic Properties of Starch-pectin Blend: A Review. Food Hydrocoll..

[17] Ji S., Xu T., Li Y., Li H., Zhong Y., Lu B. (2022). Effect of Starch Molecular Structure on Precision and Texture Properties of 3D Printed Products. Food Hydrocoll..

[18] Wang T., Liu K., Lulu Z., Yiping Z., Ling C., Xiaoxi L. (2022). Development of M Cell-Targeting Starch-Based Nanomicelles for Oral Delivery of Immunoactive Peptides. Ind. Crop. Prod..

[19] Zhang Z., Zheng B., Tang Y., Chen L. (2022). Starch Concentration is an Important Factor for Controlling Its Digestibility During Hot-Extrusion 3D Printing. Food Chem..

[20] Wang R., Li Z., Shi J., Holmes M., Wang X., Zhang J., Zhai X., Huang X., Zou X. (2021). Color 3D Printing of Pulped Yam Utilizing a Natural pH Sensitive Pigment. Addit. Manuf..

[21] Obadi M., Xu B. (2021). Review on the Physicochemical Properties, Modifications, and Applications of Starches and Its Common Modified forms Used in Noodle Products. Food Hydrocoll..

[22] Zhai X., Sun Y., Cen S., Wang X., Zhang J., Yang Z., Li Y., Wang X., Zhou C., Arslan M. (2022). Anthocyanins-Encapsulated 3D-Printable Bigels: A Colorimetric and Leaching-Resistant Volatile Amines Sensor for Intelligent Food Packaging. Food Hydrocoll..

[23] Zheng L., Liu J., Liu R., Xing Y., Jiang H. (2021). 3D Printing Performance of Gels from Wheat Starch, Flour and Whole Meal. Food Chem..

[24] Maniglia B.C., Pataro G., Ferrari G., Augusto P.E.D., Le-Bail P., Le-Bail A. (2021). Pulsed Electric Fields (PEF) Treatment to Enhance Starch 3D Printing Application: Effect on Structure, Properties, and Functionality of Wheat and Cassava Starches. Innov. Food Sci. Emerg. Technol..

[25] Chen H., Xie F., Chen L., Zheng B. (2019). Effect of Rheological Properties of Potato, Rice and Corn Starches on Their Hot-Extrusion 3D Printing Behaviors. J. Food Eng..

[26] Liu Z., Zhang M., Bhandari B., Yang C. (2018). Impact of Rheological Properties of Mashed Potatoes on 3D Printing. J. Food Eng..

[27] Liu W., Liu K., McClements D.J., Jin Z., Chen L. (2025). Fabrication and Characterization of Starch-Based Bigels Under Phase Control: Structural, Physicochemical and 3D Printing Properties. Food Hydrocoll..

[28] Qin W., Wen C.T., Zhang J.X., Dzah C.S., Zhang H.H., He Y.Q., Duan Y.Q. (2020). Structural Characterization and Physicochemical Properties of Arrowhead Resistant Starch Prepared by Different Methods. Int. J. Biol. Macromol..

[29] Feng L., Wu J., Cai L., Li M., Dai Z., Li D., Liu C., Zhang M. (2022). Effects of Different Hydrocolloids on the Water Migration, Rheological and 3D Printing Characteristics of β-Carotene Loaded Yam Starch-Based Hydrogel. Food Chem..

[30] Cui H.Y., Hu C.R., Aziz T., Albekairi T.H., Alshammari A., Lin L. (2024). Application and Evaluation of Precision in Food Ink Pattern Printing Utilizing Image-Guided Non-Planar Slicing Technology. Food Bioprocess Technol..

[31] Zhang Z.H., Zhang C., Fan Z.Y., Chen Z.J., Liu X.Y., Zhang G., Wang S.Y., Shen Y., Wang D.X., Li W.T. (2024). Development and Characterization of Whole-Grain Buckwheat Biscuit Formula Based on Extruded 3D Printing Technology. J. Food Meas. Charact..

[32] Yan D., Xu W., Yu Q., You J., Gao R., Bao Y. (2024). Pre-Rigor Salting Improves Gel Strength And Water-Holding of Surimi Gel Made from Snakehead Fish (*Channa argus*): The Role of Protein Oxidation. Food Chem..

[33] Xiong Z.Y., Shi T., Jin W.G., Bao Y.L., Monto A.R., Yuan L., Gao R.C. (2024). Gel Performance of Surimi Induced by Various Thermal Technologies: A Review. Crit. Rev. Food Sci. Nutr..

[34] Wang X., Li M.Z., Shi T., Monto A.R., Yuan L., Jin W.G., Gao R.C. (2024). Recovery of Protein-Rich Biomass from Surimi Rinsing Wastewater by Using a Sustainable cold Plasma Treatment. Food Chem. X.

[35] Xiong Z.Y., Shi T., Zhang W., Kong Y.F., Yuan L., Gao R.C. (2021). Improvement of Gel Properties of Low Salt Surimi Using Low-dose L-Arginine Combined with Oxidized Caffeic Acid. LWT-Food Sci. Technol..

[36] Guo Z., Li Z., Cen S., Liang N., Shi J., Huang X., Zou X. (2023). Preparation of *Pangasius hypophthalmus* Protein-Stabilized Pickering Emulsions and 3D Printing Application. J. Food Eng..

[37] Xiong Y., Zhao Z., Zhang N., Zhu Z., Liu Y., Zhang H., Chen W., Fan D. (2025). A Novel Method for High-Temperature Microwave 3D Printing of Golden Thread Surimi: Combination of Thermally Reversible Gelatin and κ-Carrageenan. Food Hydrocoll..

[38] Han N., Baek S., Alauddin A.A.D., Jo H., Ma Y., Lee S., Bae J.-E. (2025). Optimizing Tilapia-Based Surimi Ink for 3D Printing: Enhancing Physicochemical Properties and Printability with Ulva Powder. Food Chem..

[39] Yang R., Bao L., Liu Y., Liang J., Zheng B., Miao W., Shi X., Gao P., Zhou R., Zhao Y. (2024). Plant Oil Body as an Effective Improver for Surimi-Based 3D Printing. Addit. Manuf..

[40] Sharma R., Nath P.C., Hazarika T.K., Ojha A., Nayak P.K., Sridhar K. (2024). Recent Advances in 3D Printing Properties of Natural Food Gels: Application of Innovative Food Additives. Food Chem..

[41] Bhat Z.F., Morton J.D., Kumar S., Bhat H.F., Aadil R.M., Bekhit A.E.-D.A. (2021). 3D printing: Development of Animal Products and Special Foods. Trends Food Sci. Technol..

[42] Li X., Fan L., Liu Y., Li J. (2023). New Insights into Food O/W Emulsion Gels: Strategies of Reinforcing Mechanical Properties and Outlook of Being Applied to food 3D Printing. Crit. Rev. Food Sci. Nutr..

[43] Xu K., Wu C., Fan G., Kou X., Li X., Li T., Dou J., Zhou Y. (2023). Rheological Properties, Gel Properties and 3D Printing Performance of Soy Protein Isolate Gel Inks Added with Different Types of Apricot Polysaccharides. Int. J. Biol. Macromol..

[44] Li Z.H., Wang S.W., Qin Z., Fang W.B., Guo Z., Zou X.B. (2024). 3D Printing Properties of Heat-Induced Sodium Alginate-Whey Protein Isolate Edible Gel. Gels.

[45] Li M., Feng L., Xu Y.Y., Nie M.M., Li D.J., Zhou C.S., Dai Z.Q., Zhang Z.Y., Zhang M. (2023). Rheological Property, β-Carotene Stability and 3D Printing Characteristic of Whey Protein Isolate Emulsion Gels by Adding Different Polysaccharides. Food Chem..

[46] Yan J.K., Cai W.D., Wang C., Yu Y.B., Zhang H.N., Yang Y., Wang W.H. (2020). Macromolecular Behavior, Structural Characteristics and Rheological Properties of Alkali-Neutralization Curdlan at Different Concentrations. Food Hydrocoll..

[47] Sun W.X., Zhang R.J., Fan J., He Y., Mao X.H. (2018). Comprehensive Transformative Profiling of Nutritional and Functional Constituents During Germination of Soybean Sprouts. J. Food Meas. Charact..

[48] Cui F., Zhao S., Guan X., McClements D.J., Liu X., Liu F., Ngai T. (2021). Polysaccharide-Based Pickering Emulsions: Formation, Stabilization and Applications. Food Hydrocoll..

[49] Chevalier Y., Bolzinger M.-A. (2013). Emulsions Stabilized with Solid Nanoparticles: Pickering Emulsions. Colloids Surf. A Physicochem. Eng. Asp..

[50] Yan X., Ma C., Cui F., McClements D.J., Liu X., Liu F. (2020). Protein-Stabilized Pickering Emulsions: Formation, Stability, Properties, and Applications in Foods. Trends Food Sci. Technol..

[51] Mwangi W.W., Lim H.P., Low L.E., Tey B.T., Chan E.S. (2020). Food-Grade Pickering Emulsions for Encapsulation and Delivery of Bioactives. Trends Food Sci. Technol..

[52] Dickinson E. (2003). Hydrocolloids at Interfaces and the Influence on the Properties of Dispersed Systems. Food Hydrocoll..

[53] Fathi M., Martín Á., McClements D.J. (2014). Nanoencapsulation of Food Ingredients Using Carbohydrate Based Delivery Systems. Trends Food Sci. Technol..

[54] Melanie H., Taarji N., Zhao Y., Khalid N., Neves M.A., Kobayashi I., Tuwo A., Nakajima M. (2020). Formulation and Characterisation of O/W Emulsions Stabilised with Modified Seaweed Polysaccharides. Int. J. Food Sci. Technol..

[55] Tcholakova S., Denkov N., Lips A. (2008). Comparison of Solid Particles, Globular Proteins and Surfactants as Emulsifiers. Phys. Chem. Chem. Phys..

[56] Weiss J., Ahmad T., Zhang C., Zhang H. (2020). A Review of Recent Progress on High Internal-Phase Pickering Emulsions in Food Science. Trends Food Sci. Technol..

[57] Guo Z., Li Z.H., Cen S.Y., Liang N.N., Muhammad A., Tahir H.E., Shi J.Y., Huang X.W., Zou X.B. (2023). Modulating Hydrophilic Properties of β-Cyclodextrin/Carboxymethyl Cellulose Colloid Particles to Stabilize Pickering Emulsions for Food 3D Printing. Carbohydr. Polym..

[58] Cen S.Y., Li Z.H., Guo Z., Shi J.Y., Huang X.W., Zou X.B., Holmes M. (2023). Fabrication of Pickering Emulsions Stabilized by Citrus Pectin Modified with β-Cyclodextrin and Its Application in 3D Printing. Carbohydr. Polym..

[59] Guo Z., Yang B.X., Liang N.N., Huang X.W., Shi J.Y., Li Z.H., Paximada P., Xiaobo Z. (2025). 4D printing of Pickering emulsion: Temperature-driven color changes. J. Food Eng..

[60] Zhong Y., Wang B., Lv W., Wu Y., Lv Y., Sheng S. (2024). Recent Research and Applications in Lipid-Based Food and Lipid-Incorporated Bioink for 3D Printing. Food Chem..

[61] Cui L., Guo J., Meng Z. (2023). A Review on Food-Grade-Polymer-Based O/W Emulsion Gels: Stabilization Mechanism and 3D Printing Application. Food Hydrocoll..

[62] Chaves K.F., Barrera-Arellano D., Ribeiro A.P.B. (2018). Potential Application of Lipid Organogels for Food Industry. Food Res. Int..

[63] Pérez B., Nykvist H., Brøgger A.F., Larsen M.B., Falkeborg M.F. (2019). Impact of Macronutrients Printability and 3D-Printer Parameters on 3D-Food Printing: A Review. Food Chem..

[64] Portanguen S., Tournayre P., Sicard J., Astruc T., Mirade P.-S. (2019). Toward the Design of Functional Foods and Biobased Products by 3D Printing: A Review. Trends Food Sci. Technol..

[65] Johannesson J., Wu M., Johansson M., Bergström C.A. (2023). Quality Attributes for Printable Emulsion Gels and 3D-Printed Tablets: Towards Production of Personalized Dosage Forms. Int. J. Pharm..

[66] Lanaro M., Forrestal D.P., Scheurer S., Slinger D.J., Liao S., Powell S.K., Woodruff M.A. (2017). 3D Printing Complex Chocolate Objects: Platform Design, Optimization and Evaluation. J. Food Eng..

[67] Mantihal S., Prakash S., Godoi F.C., Bhandari B. (2017). Optimization of Chocolate 3D printing by Correlating Thermal and Flow Properties with 3D Structure Modeling. Innov. Food Sci. Emerg. Technol..

[68] Parid D.M., Talib A.T., Baharuddin A.S., Abdul Rahman N.A., Mohammed M.A.P., Wakisaka M. (2025). Mechanics of 3D Printing Process of White Chocolate. J. Food Eng..

[69] Wang D., Guo J., Wang Y., Yang Y., Jiang B., Li D., Feng Z., Liu C. (2023). Whey Protein Isolate Nanofibrils as Emulsifying Agent to Improve Printability of Cheddar Cheese for 3D Printing. Food Hydrocoll..

[70] Ross M.M., Crowley S.V., Crotty S., Oliveira J., Morrison A.P., Kelly A.L. (2021). Parameters Affecting the Printability of 3D-Printed Processed Cheese. Innov. Food Sci. Emerg. Technol..

[71] In J., Jeong H., Song S., Min S.C. (2021). Determination of Material Requirements for 3D Gel Food Printing Using a Fused Deposition Modeling 3D Printer. Foods.

[72] Cui Y., Li C., Guo Y., Liu X., Zhu F., Liu Z., Liu X., Yang F. (2022). Rheological & 3D Printing Properties of Potato Starch Composite Gels. J. Food Eng..

[73] Mahmood K., Kamilah H., Shang P.L., Sulaiman S., Ariffin F., Alias A.K. (2017). A review: Interaction of Starch/Non-Starch Hydrocolloid Blending and the Recent Food Applications. Food Biosci..

[74] Wang S., Copeland L. (2013). Molecular Disassembly of Starch Granules During Gelatinization and Its Effect On Starch Digestibility: A Review. Food Funct..

[75] Siddiqui S.A., Alvi T., Biswas A., Shityakov S., Gusinskaia T., Lavrentev F., Dutta K., Khan M.K.I., Stephen J., Radhakrishnan M. (2023). Food Gels: Principles, Interaction Mechanisms and Its Microstructure. Crit. Rev. Food Sci. Nutr..

[76] Pereira R.N., Rodrigues R., Avelar Z., Leite A.C., Leal R., Pereira R.S., Vicente A. (2024). Electrical Fields in the Processing of Protein-Based Foods. Foods.

[77] Temkov M., Mureșan V. (2021). Tailoring the Structure of Lipids, Oleogels and Fat Replacers by Different Approaches for Solving the Trans-Fat Issue—A Review. Foods.

[78] Wang W., Sun B., Deng J., Ai N. (2024). Addressing Flavor Challenges in Reduced-Fat Dairy Products: A Review from the Perspective of Flavor Compounds and Their Improvement Strategies. Food Res. Int..

[79] Dong Z., Levkin P.A. (2023). 3D Microprinting of Super-Repellent Microstructures: Recent Developments, Challenges, and Opportunities. Adv. Funct. Mater..

[80] Liu Y., Yu Y., Liu C., Regenstein J.M., Liu X., Zhou P. (2019). Rheological and Mechanical Behavior of Milk Protein Composite Gel for Extrusion-Based 3D Food Printing. LWT.

[81] Chen Y., Zhang M., Sun Y., Phuhongsung P. (2022). Improving 3D/4D Printing Characteristics of Natural Food Gels by Novel Additives: A Review. Food Hydrocoll..

[82] Nachal N., Moses J., Karthik P., Anandharamakrishnan C. (2019). Applications of 3D Printing in Food Processing. Food Eng. Rev..

[83] Pereira T., Barroso S., Gil M.M. (2021). Food Texture Design by 3D Printing: A Review. Foods.

[84] Dankar I., Haddarah A., Omar F.E., Sepulcre F., Pujolà M. (2018). 3D Printing Technology: The New Era for Food Customization and Elaboration. Trends Food Sci. Technol..

[85] Escalante-Aburto A., Trujillo-de Santiago G., Álvarez M.M., Chuck-Hernández C. (2021). Advances and Prospective Applications of 3D Food Printing for Health Improvement and Personalized Nutrition. Compr. Rev. Food Sci. Food Saf..

[86] Xie Y., Liu Q., Zhang W., Yang F., Zhao K., Dong X., Prakash S., Yuan Y. (2023). Advances in the Potential Application of 3D Food Printing to Enhance Elderly Nutritional Dietary Intake. Foods.

[87] Mann T., Heuberger R., Wong H. (2013). The Association Between Chewing and Swallowing Difficulties and Nutritional Status in Older Adults. Aust. Dent. J..

[88] DeMatteo C., Matovich D., Hjartarson A. (2005). Comparison of Clinical and Videofluoroscopic Evaluation of Children with Feeding and Swallowing Difficulties. Dev. Med. Child Neurol..

[89] Lipton J.I., Cutler M., Nigl F., Cohen D., Lipson H. (2015). Additive Manufacturing for the Food Industry. Trends Food Sci. Technol..

[90] Dankar I., Haddarah A., El Omar F., Sepulcre F., Pujolà M. (2018). Assessing the Microstructural and Rheological Changes Induced by Food Additives on Potato Puree. Food Chem..

[91] Zhu J., Yang Y., Qiao S., Dai H., Chen H., Fu Y., Ma L., Wang H., Zhang Y. (2024). 4D Printing of Betanin/Gelatin/Nano-Chitin Complexes-Functionalized Surimi via Disulfide Bonds, and Its Applicability in Dysphagia Diets. Food Hydrocoll..

[92] Xu B.G., Wang X.D., Chitrakar B., Xu Y., Wei B.X., Wang B., Lin L., Guo Z.M., Zhou C.S., Ma H.L. (2025). Effect of Various Physical Modifications of Pea Protein Isolate (PPI) on 3D Printing Behavior and Dysphagia Properties of Strawberry-PPI gels. Food Hydrocoll..

[93] Guan T., Ren C., Feng Y., Gao Y., Wang Q., Rao S., Xiao L., Yang Z., Liu Q. (2024). Implementation of Succinylated Lactoferrin-Luteolin Nanocomplex-Based 3D Printing Inks in Nutritional and Textural Customization for Dysphagia Diets: Printing Mechanism, Improved Bioactivity and In Vitro Bioaccessibility. LWT.

[94] Wu H., Sang S., Weng P., Pan D., Wu Z., Yang J., Liu L., Farag M.A., Xiao J., Liu L. (2023). Structural, Rheological, and Gelling Characteristics of Starch-Based Materials in Context to 3D Food Printing Applications in Precision Nutrition. Compr. Rev. Food Sci. Food Saf..

[95] Prendergast M.E., Burdick J.A. (2020). Recent Advances in Enabling Technologies in 3D Printing for Precision Medicine. Adv. Mater..

[96] Thorakkattu P., Awasti N., Sajith Babu K., Khanashyam A.C., Deliephan A., Shah K., Singh P., Pandiselvam R., Nirmal N.P. (2024). 3D printing: Trends and Approaches Toward Achieving Long-Term Sustainability in the Food Industry. Crit. Rev. Biotechnol..

[97] Lu X., Qian S., Wu X., Lan T., Zhang H., Liu J. (2024). Research Progress of Protein Complex Systems and Their Application in Food: A Review. Int. J. Biol. Macromol..

[98] Ainis W.N., Feng R., van den Berg F.W., Ahrné L. (2023). Comparing the Rheological and 3D Printing Behavior of Pea and Soy Protein Isolate Pastes. Innov. Food Sci. Emerg. Technol..

[99] Shi H., Li J., Xu E., Yang H., Liu D., Yin J. (2023). Microscale 3D Printing of Fish Analogues Using Soy Protein Food Ink. J. Food Eng..

[100] Wang J., Goyanes A., Gaisford S., Basit A.W. (2016). Stereolithographic (SLA) 3D printing of Oral Modified-Release Dosage Forms. Int. J. Pharm..

[101] Goyanes A., Det-Amornrat U., Wang J., Basit A.W., Gaisford S. (2016). 3D Scanning and 3D Printing as Innovative Technologies for Fabricating Personalized Topical Drug Delivery Systems. J. Control. Release.

[102] Derossi A., Caporizzi R., Oral M., Severini C. (2020). Analyzing the Effects of 3D Printing Process per se on the Microstructure and Mechanical Properties of Cereal Food Products. Innov. Food Sci. Emerg. Technol..

[103] Khan R.S., Grigor J., Winger R., Win A. (2013). Functional Food Product Development–Opportunities and Challenges for Food Manufacturers. Trends Food Sci. Technol..

[104] Kurapkienė A., Vinauskienė R., Jasutienė I., Keršienė M., Damulevičienė G., Knašienė J., Lesauskaitė V., Sulmont-Rossé C., Eisinaitė V., Leskauskaitė D. (2024). Bigel as a Curcumin Delivery System And Its Application in 3D-Printed in-Between-Meal Foods to Boost the Immune System of Elderly People. Food Biosci..

[105] Tomašević I., Putnik P., Valjak F., Pavlić B., Šojić B., Markovinović A.B., Kovačević D.B. (2021). 3D Printing as Novel Tool for Fruit-Based Functional Food Production. Curr. Opin. Food Sci..

[106] Sundarsingh A., Zhang M., Mujumdar A.S., Li J. (2024). Research Progress in Printing Formulation for 3D Printing of Healthy Future Foods. Food Bioprocess Technol..

[107] Murugan M., Ramasamy S.K., Venkatesan G., Lee J., Barathi S., Kandasamy S., Sarangi P.K. (2024). The Comprehensive Review on 3D Printing-Pharmaceutical Drug Delivery and Personalized Food and Nutrition. Food Chem..

[108] Derossi A., Spence C., Corradini M.G., Jekle M., Fahmy A.R., Caporizzi R., Devahastin S., Moses J.A., Le-Bail A., Zhou W. (2024). Personalized, Digitally Designed 3D Printed Food Towards the Reshaping of Food Manufacturing and Consumption. npj Sci. Food.

[109] Tibbits S. (2014). 4D Printing: Multi-Material Shape Change. Archit. Des..

[110] Gebhardt A., Hötter J.-S., Gebhardt A., Hötter J.-S. (2016). 2—Characteristics of the Additive Manufacturing Process. Additive Manufacturing.

[111] Navaf M., Sunooj K.V., Aaliya B., Akhila P.P., Sudheesh C., Mir S.A., George J. (2022). 4D Printing: A New Approach for Food Printing; Effect of Various Stimuli on 4D Printed Food Properties. A Comprehensive Review. Appl. Food Res..

[112] Shinde S., Mane R., Vardikar A., Dhumal A., Rajput A. (2023). 4D Printing: From Emergence to Innovation Over 3D Printing. Eur. Polym. J..

[113] Piqueras-Fiszman B., Alcaide J., Roura E., Spence C. (2012). Is It the Plate or Is It the Food? Assessing the Influence of the Color (Black or White) and Shape of the Plate on the Perception of the Food Placed on it. Food Qual. Prefer..

[114] He C., Zhang M., Guo C. (2020). 4D Printing of Mashed Potato/Purple Sweet Potato Puree with Spontaneous Color Change. Innov. Food Sci. Emerg. Technol..

[115] Cen S., Meng Z. (2024). 4D Printing: A Novel Application for Structuring Oils with Fat-Analog Characteristics. Trends Food Sci. Technol..

[116] Oral M.O., Derossi A., Caporizzi R., Severini C. (2021). Analyzing the Most Promising Innovations in Food Printing. Programmable Food Texture and 4D Foods. Future Foods.

[117] Cheng Y., Fu Y., Ma L., Yap P.L., Losic D., Wang H., Zhang Y. (2022). Rheology of Edible Food Inks from 2D/3D/4D Printing, and its Role in Future 5D/6D Printing. Food Hydrocoll..

[118] Zhang H., Huang S., Sheng J., Fan L.S., Zhou J.Z., Shan M.Y., Wei J.A., Wang C., Yang H.W., Lu J.Z. (2022). 4D Printing of Ag Nanowire-Embedded Shape Memory Composites with Stable and Controllable Electrical Responsivity: Implications for Flexible Actuators. ACS Appl. Nano Mater..

[119] Beaudoin A. (2016). JMS-1704: Multihead 3D Printer.

[120] Park J.-H., Jang J.-S. (2020). FDM Full Color 3D Printer GUI Design Development of Checklist for Usability. J. Korea Converg. Soc..

[121] Chen Y., Zhang W.J., Zhao T., Li F., Zhang M., Li J., Zou Y., Wang W., Cobbina S.J., Wu X.Y. (2016). Adsorption Properties of Macroporous Adsorbent Resins for Separation of Anthocyanins from Mulberry. Food Chem..

[122] Zhang H.N., Ma Y.K. (2017). Optimisation of High Hydrostatic Pressure Assisted Extraction of Anthocyanins from Rabbiteye Blueberry Pomace. Czech J. Food Sci..

[123] Tian X.Y., Aheto J.H., Bai J.W., Dai C.X., Ren Y., Chang X.H. (2021). Quantitative Analysis and Visualization of Moisture and Anthocyanins Content in Purple Sweet Potato by Vis-NIR Hyperspectral Imaging. J. Food Process. Preserv..

[124] Herrera-Balandrano D.D., Chai Z., Beta T., Feng J., Huang W.Y. (2021). Blueberry Anthocyanins: An Updated Review on Approaches to Enhancing Their Bioavailability. Trends Food Sci. Technol..

[125] Wang L., Yang S.F., Yang Y.Y., Jiang H.Z., Huang W.Y., Bian Y.Y., Li B. (2024). Effects of Endogenous Anthocyanins from Purple corn on the Quality, Physicochemical Properties and Antioxidant Capacity of Bread. J. Food Meas. Charact..

[126] Zhai X., Xue Y., Song W., Sun Y., Shen T., Zhang X., Li Y., Zhang D., Zhou C., Zhang J. (2024). Rapid and Facile Synthesis of Homoporous Colorimetric Films Using Leaf Vein-Mediated Emulsion Evaporation Method for Visual Monitoring of Food Freshness. J. Agric. Food Chem..

[127] Hashim S.B.H., Tahir H.E., Lui L., Zhang J.J., Zhai X.D., Mahdi A.A., Ibrahim N.A., Mahunu G.K., Hassan M.M., Xiaobo Z. (2023). Smart films of Carbohydrate-Based/Sunflower Wax/Purple Chinese Cabbage Anthocyanins: A Biomarker of Chicken Freshness. Food Chem..

[128] Ai J., Wu Q., Battino M., Bai W., Tian L. (2021). Using Untargeted Metabolomics to Profile the Changes in Roselle (*Hibiscus sabdariffa* L.) Anthocyanins During Wine Fermentation. Food Chem..

[129] Gao Q.C., Li Y., Li Y.H., Liang Y., Zhang Z.Y. (2022). Profile of Anthocyanins in Purple Vegetables Commonly Consumed in China and Their Relationship with Antioxidant Abilities. J. Food Meas. Charact..

[130] Herrera-Balandrano D.D., Wang J., Chai Z., Zhang X., Wang J., Wang N., Huang W. (2023). Impact of In Vitro Gastrointestinal Digestion on Rabbiteye Blueberry Anthocyanins and Their Absorption Efficiency in Caco-2 Cells. Food Biosci..

[131] Chen Z., Ma J., Li P., Wen B., Wang Y., Ma Y., Huang W. (2023). Preparation of Hypoglycemic Anthocyanins from Mulberry (*Fructus mori*) Fruits by Ultrahigh Pressure Extraction. Innov. Food Sci. Emerg. Technol..

[132] Chen Y., Li Q., Zhao T., Zhang Z., Mao G.H., Feng W.W., Wu X.Y., Yang L.Q. (2017). Biotransformation and Metabolism of Three Mulberry Anthocyanin Monomers by Rat Gut Microflora. Food Chem..

[133] Zhai X.D., Shi J.Y., Zou X.B., Wang S., Jiang C.P., Zhang J.J., Huang X.W., Zhang W., Holmes M. (2017). Novel Colorimetric Films Based on Starch/Polyvinyl Alcohol Incorporated with Roselle Anthocyanins for Fish Freshness Monitoring. Food Hydrocoll..

[134] Zhang J.J., Zou X.B., Zhai X.D., Huang X.W., Jiang C.P., Holmes M. (2019). Preparation of an Intelligent pH Film Based on Biodegradable Polymers and Roselle Anthocyanins for Monitoring Pork Freshness. Food Chem..

[135] Zhang J.J., Huang X.W., Shi J.Y., Liu L., Zhang X.A., Zou X.B., Xiao J.B., Zhai X.D., Zhang D., Li Y.X. (2021). A Visual Bi-layer Indicator Based on Roselle Anthocyanins with High Hydrophobic Property for Monitoring Griskin Freshness. Food Chem..

[136] Kwaw E., Ma Y.K., Tchabo W., Apaliya M.T., Wu M., Sackey A.S., Xiao L.L., Tahir H.E. (2018). Effect of *Lactobacillus* Strains on Phenolic Profile, Color Attributes and Antioxidant Activities of Lactic-Acid-Fermented Mulberry Juice. Food Chem..

[137] Zhai X.D., Wang X.Y., Zhang J.J., Yang Z.K., Sun Y., Li Z.H., Huang X.W., Holmes M., Gong Y.Y., Povey M. (2020). Extruded Low Density Polyethylene-Curcumin Film: A Hydrophobic Ammonia Sensor for Intelligent Food Packaging. Food Packag. Shelf Life.

[138] Zhou C.K., Huang C.Q., Li L., Tian Y.N., Zhang J., Lin L., Li C.Z., Ye Y. (2024). Apricot Polysaccharides as New Carriers to Make Curcumin Nanoparticles and Improve Its Stability and Antibacterial Activity. J. Food Sci..

[139] Hamadou A.H., Zhang J.Y., Xu B. (2024). Strategies to Protect and Deliver Curcumin via Zein Nanocomposites for Food Applications. Food Rev. Int..

[140] Wang H.P., Gao S.M., Zhang D., Wang Y.L., Zhang Y., Jiang S.S., Li B., Wu D.X., Lv G.H., Zou X.B. (2022). Encapsulation of Catechin or Curcumin in co-Crystallized Sucrose: Fabrication, Characterization and Application in Beef Meatballs. LWT-Food Sci. Technol..

[141] Li C.Y., Liu D., Huang M.G., Huang W.Y., Li Y., Feng J. (2022). Interfacial Engineering Strategy to Improve the Stabilizing Effect of Curcumin-Loaded Nanostructured Lipid Carriers. Food Hydrocoll..

[142] Navarro-Hortal M.D., Romero-Márquez J.M., Jiménez-Trigo V., Xiao J.B., Giampieri F., Forbes-Hernández T.Y., Grosso G., Battino M., Sánchez-González C., Quiles J.L. (2023). Molecular Bases for the Use of Functional Foods in the Management of Healthy Aging: Berries, Curcumin, Virgin Olive Oil and Honey; Three Realities and a Promise. Crit. Rev. Food Sci. Nutr..

[143] Yan J.K., Qiu W.Y., Wang Y.Y., Wu J.Y. (2017). Biocompatible Polyelectrolyte Complex Nanoparticles from Lactoferrin and Pectin as Potential Vehicles for Antioxidative Curcumin. J. Agric. Food Chem..

[144] Wang H.P., Li Z.H., Meng Y., Lv G.H., Wang J.P., Zhang D., Shi J.Y., Zhai X.D., Meng X.R., Zou X.B. (2024). Co-Delivery Mechanism of Curcumin/Catechin Complex by Modified Soy Protein Isolate: Emphasizing Structure, Functionality, and Intermolecular Interaction. Food Hydrocoll..

[145] Kang L.X., Liang Q.F., Abdul Q., Rashid A., Ren X.F., Ma H.L. (2023). Preparation Technology and Preservation Mechanism of γ-CD-MOFs Biaological Packaging Film Loaded with Curcumin. Food Chem..

[146] Zhang J., Huang X., Zou X., Shi J., Zhai X., Liu L., Li Z., Holmes M., Gong Y., Povey M. (2021). A Visual Indicator Based on Curcumin with High Stability for Monitoring the Freshness of Freshwater Shrimp, *Macrobrachium rosenbergii*. J. Food Eng..

[147] Feng J., Wang Z., Huang W.Y., Zhao X.Y., Xu L.J., Teng C., Li Y. (2025). Hyaluronic Acid-Decorated Lipid Nanocarriers as Novel Vehicles for Curcumin: Improved Stability, Cellular Absorption, and Anti-Inflammatory Effects. Food Chem..

[148] Shen C., Chen W., Li C., Chen X., Cui H., Lin L. (2023). 4D Printing System Stimulated by Curcumin/Whey Protein Isolate Nanoparticles: A Comparative Study of Sensitive Color Change and Post-Processing. J. Food Eng..

[149] Cen S.Y., Li Z.H., Guo Z.A., Li H.R., Shi J.Y., Huang X.W., Zou X.B., Holmes M. (2022). 4D Printing of a Citrus Pectin/β-CD Pickering Emulsion: A Study on Temperature Induced Color Transformation. Addit. Manuf..

[150] Lin Q., Hu Y., Qiu C., Li X., Sang S., McClements D.J., Chen L., Long J., Xu X., Wang J. (2023). Peanut Protein-Polysaccharide Hydrogels Based on Semi-Interpenetrating Networks Used for 3D/4D Printing. Food Hydrocoll..

[151] Ghazal A.F., Zhang M., Guo Z. (2023). Microwave-Induced Rapid 4D Change in Color of 3D Printed Apple/Potato Starch Gel with Red Cabbage Juice-Loaded WPI/GA Mixture. Food Res. Int..

[152] Chen C., Zhang M., Guo C., Chen H. (2021). 4D Printing of Lotus Root Powder Gel: Color Change Induced by Microwave. Innov. Food Sci. Emerg. Technol..

[153] Xiao K., Zhang Y., Pan L., Tu K. (2024). Study on Color and Flavor Changes of 4D Printed White Mushroom Gel with Microcapsules Containing Gelatin/β-Cyclodextrin Induced by Microwave Heating. Int. J. Biol. Macromol..

[154] Gu G., Cui Z., Du X., He P., Rong C., Tao H., Wei G., Xi Y. (2024). Recent Advances in Biomacromolecule-Reinforced 2D Material (2DM) Hydrogels: From Interactions, Synthesis, and Functionalization to Biomedical Applications. Adv. Funct. Mater..

[155] Angioloni A., Collar C. (2009). Small and Large Deformation Viscoelastic Behaviour of Selected Fibre Blends with Gelling Properties. Food Hydrocoll..

[156] Ding Q., Wu Z., Tao K., Wei Y., Wang W., Yang B.-R., Xie X., Wu J. (2022). Environment Tolerant, Adaptable and Stretchable Organohydrogels: Preparation, Optimization, and Applications. Mater. Horiz..

[157] Cao Y., Mezzenga R. (2020). Design principles of food gels. Nat. Food.

[158] Chen F., Zhang M., Liu Z., Bhandari B. (2021). 4D Deformation Based on Double-Layer Structure of the Pumpkin/Paper. Food Struct..

[159] Noree S., Pinyakit Y., Tungkijanansin N., Kulsing C., Hoven V.P. (2023). Shape Transformation of 4D Printed Edible Insects Triggered by Thermal Dehydration. J. Food Eng..

[160] Jiang Q., Binks B.P., Meng Z. (2022). Double Scaffold Networks Regulate Edible Pickering Emulsion Gel for Designing Thermally Actuated 4D Printing. Food Hydrocoll..

[161] He C., Zhang M., Devahastin S. (2021). Microwave-Induced Deformation Behaviors of 4D Printed Starch-Based Food Products as Affected by Edible Salt and Butter Content. Innov. Food Sci. Emerg. Technol..

[162] Lv Y., Wang B., Cheng Y., Lv W., Zeng S., Xiao H. (2024). Printing Characteristics and Microwave Infrared-Induced 4D Printing of Chestnut Powder Composite Paste. J. Food Eng..

